# Insights Into the Bovine Milk Microbiota in Dairy Farms With Different Incidence Rates of Subclinical Mastitis

**DOI:** 10.3389/fmicb.2018.02379

**Published:** 2018-10-16

**Authors:** Maoda Pang, Xing Xie, Hongduo Bao, Lichang Sun, Tao He, Hang Zhao, Yan Zhou, Lili Zhang, Hui Zhang, Ruicheng Wei, Kaizhou Xie, Ran Wang

**Affiliations:** ^1^Key Laboratory of Control Technology and Standard for Agro-product Safety and Quality, Jiangsu Key Laboratory for Food Quality and Safety-State Key Laboratory Cultivation Base of Ministry of Science and Technology, Institute of Food Safety and Nutrition, Jiangsu Academy of Agricultural Sciences, Nanjing, China; ^2^Key Laboratory of Veterinary Biological Engineering and Technology, Institute of Veterinary Medicine, Ministry of Agriculture, Jiangsu Academy of Agricultural Sciences, Nanjing, China; ^3^Key Laboratory for Animal Genetics, Breeding, Reproduction and Molecular Design of Jiangsu Province, College of Animal Science and Technology, Yangzhou University, Yangzhou, China

**Keywords:** milk microbiota, 16S rRNA gene sequencing, bovine mastitis, IRSCM, mastitis-causing pathogens

## Abstract

Bovine mastitis continues to be a complex disease associated with significant economic loss in dairy industries worldwide. The incidence rate of subclinical mastitis (IRSCM) can show substantial variation among different farms; however, the milk microbiota, which have a direct influence on bovine mammary gland health, have never been associated with the IRSCM. Here, we aimed to use high-throughput DNA sequencing to describe the milk microbiota from two dairy farms with different IRSCMs and to identify the predominant mastitis pathogens along with commensal or potential beneficial bacteria. Our study showed that *Klebsiella*, *Escherichia–Shigella*, and *Streptococcus* were the mastitis-causing pathogens in farm A (with a lower IRSCM), while *Streptococcus* and *Corynebacterium* were the mastitis-causing pathogens in farm B (with a higher IRSCM). The relative abundance of all pathogens in farm B (22.12%) was higher than that in farm A (9.82%). However, the genus *Bacillus* was more prevalent in farm A. These results may be helpful for explaining the lower IRSCM in farm A. Additionally, the gut-associated genera *Prevotella*, *Ruminococcus*, *Bacteroides*, *Rikenella*, and *Alistipes* were prevalent in all milk samples, suggesting gut bacteria can be one of the predominant microbial contamination in milk. Moreover, *Listeria monocytogenes* (a foodborne pathogen) was found to be prevalent in farm A, even though it had a lower IRSCM. Overall, our study showed complex diversity between the milk microbiota in dairy farms with different IRSCMs. This suggests that variation in IRSCMs may not only be determined by the heterogeneity and prevalence of mastitis-causing pathogens but also be associated with potential beneficial bacteria. In the future, milk microbiota should be considered in bovine mammary gland health management. This would be helpful for both the establishment of a targeted mastitis control system and the control of the safety and quality of dairy products.

## Introduction

Mastitis, defined as inflammation of the mammary gland, is commonly associated with bacterial infection (McDougall et al., 2009). Bovine mastitis continues to be one of the major veterinary and economic issues that affects dairy industries worldwide (Ruegg, 2017). It is well known that the incidence rate of subclinical mastitis (IRSCM) can show substantial variation among different dairy farms, and the predominant mastitis-causing pathogens can also differ considerably (Olde Riekerink et al., 2008). To develop successful control programs for mastitis, it is very important to identify the predominant mastitis-causing pathogens (Ruegg, 2017). However, there were no bacteria detected by conventional approaches in 20–40% of milk samples from mastitis cases due to the low concentration of bacteria or to the fastidious nutritional and growth requirements (Taponen et al., 2009). In addition, recent studies suggested that mastitis may be associated not only with the mastitis pathogens but also with an imbalance of the milk microbiota (Oikonomou et al., 2012; Kuehn et al., 2013). From another perspective, milk microbiota can directly affect subsequent development of dairy products. Therefore, to establish a targeted mastitis control system and to improve the safety and quality of dairy products, it is of great importance to understand the bacterial community present in milk.

Over the last two decades, methods such as real-time PCR (Katholm et al., 2012; Holmoy et al., 2018), multiplex PCR (mPCR) (Shome et al., 2011), and denaturing gradient gel electrophoresis (DGGE) PCR (Kuang et al., 2009) have been used to identify bacterial DNA in milk samples. In recent years, increasing evidence has shown that sequencing of the 16S rRNA gene can identify almost the entire bacterial community, both commensal and pathogenic, since it can overcome the limitations of the culture-based bacterial detection method (Kennedy et al., 2016). Few studies have been carried out to understand the diversity of microbiota in healthy and mastitic milk samples (Kuehn et al., 2013; Oikonomou et al., 2014; Oultram et al., 2017). Previous studies reported significant differences between the microbiota of milk from healthy and mastitic quarters (Kuehn et al., 2013). Furthermore, samples derived from healthy quarters could be easily discriminated from samples derived from clinical mastitis and culture negative quarters based on their microbiota profiles (Oikonomou et al., 2014). Oultram et al. (2017) reported that 16S rRNA gene sequencing can be used to diagnose clinical mastitis, and *Streptococcus* *uberis* and *Staphylococcus* are identified in most cattle. In addition, sequence-based microbiota analyses were also used to understand the microbial diversity of feces (Oikonomou et al., 2013) and teats (Falentin et al., 2016), to assess the impact of transfer to a milk processing facility (Kable et al., 2016), and to identify possible sources of raw milk contamination (Doyle et al., 2017b). However, no study has been conducted to compare the microbiota in milk samples from different dairy farms, especially from farms with different IRSCMs.

Many studies have been conducted to determine the association between risk factors and the IRSCM; many risk factors, including age of the bovine, body condition score, stage of lactation, mammary regression, management practices, herd housing, milking machine, nutrition, weather and climate have been reported to be associated with the IRSCM (Olde Riekerink et al., 2008; Santman-Berends et al., 2016). However, the milk microbiota, which can have a direct impact on bovine mammary gland health, has never been associated with the IRSCM. Therefore, the specific objectives of this study were to use high-throughput DNA sequencing to investigate the milk microbiota from two dairy farms with different IRSCMs and to identify the predominant mastitis pathogens and the commensal or potential beneficial bacteria.

## Materials and Methods

### Sample Collection

Milk samples were collected from commercial dairy farms A (66 samples) and B (72 samples), which both located in Nanjing, Jiangsu province, China. The cows were all Holstein dairy cattle and were on twice-daily milking. The IRSCMs of farm A and farm B determined by herd veterinarians were 16.9% (306/1860) and 56.2% (930/1656), respectively. Two milk collections were obtained from 4-year-old cows at 25 and 26 May 2016, separately. Each milk sample was collected from one teat per cow. To exclude the effect of antibiotics on milk microbiota, the milk samples were collected from cows that had not been treated with antibiotics for at least 1 month. The sampling procedure was performed during the afternoon milking according to a previous study (Schukken et al., 2009). Briefly, the bovine teats were thoroughly washed with osmosis water and 70% ethanol and dried using individual paper towels. Then, the first three streams of milk were discarded, and the milk samples (about 30 mL) were collected using 50 mL sterile plastic tubes. In this study, the subclinical mastitis status was evaluated using milk somatic cell counts (SCC) calculated by Fossomatic 5000TM automatic equipment (Foss Electric, Hillerød, Denmark). Subclinical mastitis was suspected when the SCC were greater than 500,000 cells/mL, but with no inflammation of the udder (Shittu et al., 2012; Kulkarni and Kaliwal, 2013). Cows were considered healthy when the SCC were lower than 100,000 cells/mL with no inflammation of the udder (Shittu et al., 2012; Kulkarni and Kaliwal, 2013). The samples were transferred to the laboratory using a mobile refrigerator and stored at -70°C for later analysis. For comparison purposes, 32 milk samples collected from farm A (16 samples from healthy cows and 16 samples from cows with subclinical mastitis) and 32 milk samples collected from farm B (16 samples from healthy cows and 16 samples from cows with subclinical mastitis) were further analyzed.

### DNA Extraction and High-Throughput Sequencing

To extract microbial DNA, 1.8 mL of each milk sample was centrifuged at 10000 *g* for 10 min to generate a pellet from which DNA was extracted using the Powerfood Microbial DNA Isolation kit (Mo Bio Laboratories Inc., Carlsbad, CA, United States) according to the manufacturer’s instructions. The DNA was then used as a template for polymerase chain reaction. The V1–V2 region of the bacterial 16S rRNA genes from each sample was amplified using the universal primer set 27F and 338R (Oikonomou et al., 2014), which contained an 8-base unique barcode that was used to tag the PCR products from each sample. After the quantification, qualification and purification of the PCR products, a sequencing library was generated using NEB Next^®^ Ultra^TM^ DNA Library Prep Kit for Illumina (NEB, Ipswich, MA, United States). Finally, the library was sequenced using an Illumina HiSeq 2000 system (Illumina, Inc., San Diego, CA, United States), and 300 bp paired-end reads were generated. The reads were deposited in the National Center for Biotechnology Information (NCBI) Sequence Read Archive (SRA) with accession number SRP149195.

### Bioinformatic Analysis

Paired-end reads were merged using FLASH (Magoc and Salzberg, 2011) and the effective reads were obtained after removing the chimeric sequences (Edgar et al., 2011). Sequences were analyzed by the UPARSE software package using the UPARSE-OTU and UPARSE-OUT ref algorithms (Edgar, 2013) and were assigned to the same operational taxonomic units (OTUs) with ≥97% similarity. The representative sequence for each OTU was selected and annotated for taxonomic information using QIIME (Caporaso et al., 2010) and the Ribosomal Database Project classifier (Cole et al., 2009). To facilitate comparison, the milk samples were classified into eight groups according to their origin: group AM (16 samples from mastitic cows in farm A), group AH (16 samples from healthy cows in farm A), group BM (16 samples from mastitic cows in farm B), group BH (16 samples from healthy cows in farm B), group A (which consisted of groups AM and AH), group B (which consisted of groups AH and BH). The number of obtained reads and the percent of the sequence that could be annotated for different taxonomic levels was calculated for each group. Subsequently, the ten most abundant microbial phyla and the predominant genera whose abundance was higher than 0.5% in at least one group were identified. A two-way hierarchically clustered heatmap of the bacterial distribution in different milk samples was conducted using ClustVis based on average linkage clustering and Euclidean distance (Metsalu and Vilo, 2015). To calculate the genus diversity within individual groups, Perl scripts packaged in QIIME (Caporaso et al., 2010) were used to analyze the alpha diversity (Shannon index). To compare similarities within the whole community, principal component analysis (PCA) based on weighted UniFrac distances was also conducted using Perl scripts packaged in QIIME (Caporaso et al., 2010). Linear discriminant analysis (LDA) effect size (LEfSe) (Segata et al., 2011) was used to assess the microbial compositional differences between the two groups of samples at the genus or higher taxonomic level. The Spearman’s correlation coefficient values of each pair of predominant genera were calculated using SPSS Statics (version 22.0, SPSS Inc.). To reduce false-positives caused by excessive mutual exclusions, the genera that existed in less than half of the milk samples were removed (Li et al., 2015). The co-occurrence network analysis and visualization were conducted by the Gephi open source graph visualization software tool (version 0.9.2), using the force-directed algorithm ForceAtlas2 (Bastian et al., 2009).

### Statistical Analysis

Data were collected and analyzed using SPSS Statics (version 22.0, SPSS Inc., Chicago, IL, United States). The diversity among different groups was analyzed by analysis of variance (ANOVA), followed by Tukey’s and Dunnett’s T3 multiple comparison tests. In these analyses, *P* < 0.05 was considered to be statistically significant, while *P* < 0.01 was considered to be an extremely significant difference.

## Results

### Sequencing Results

**FIGURE 1:**
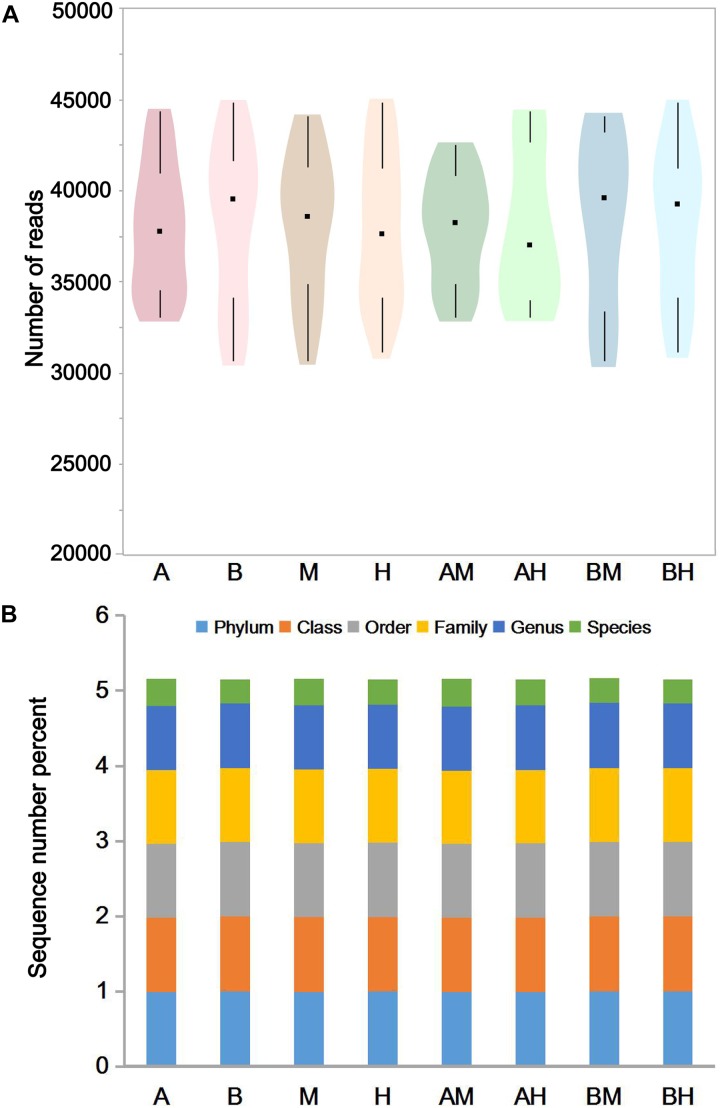
The number of effective reads in each group **(A)** and taxonomic classification of the effective reads at different levels **(B)**. Violin plots show the distribution of the number of effective reads in each group, and the black points represent the medians of the number of effective reads **(A)**. The colors represent the sequence number percent of effective reads annotated to this taxonomic level **(B)**.

The pyrosequencing of milk samples generated a total of 2,585,190 reads, of which 2,396,471 (92.7%) effective reads were ultimately analyzed by the RDP classifier after exclusion due to trimming and quality control. The number of effective reads per sample ranged from 30,693 to 44,837 (median 38,358; mean 38,089). The milk samples were classified into groups A, B, M, H, AM, AH, BM, and BH according to their origin. As shown in **Figure 1A**, the violin plots showed the distribution of the number of effective reads in each group. The medians of the number of effective reads in group A, B, M, H, AM, AH, BM, and BH were 37767, 39553, 38577, 37585, 38227, 36982, 39577, and 39274, respectively. No significant differences were observed among the number of effective reads of different groups. These data demonstrate that the relative abundance could be used in the following study. As shown in **Figure 1B**, more than 97.5% of the sequences in each group could be assigned to the level of phyla, class, order and family. Additionally, more than 85.4% of the sequences in each group could be assigned to the level of genus, and the percentage of sequences assigned to the level of species ranged from 31.6 to 36.8%.

### Microbiota of Milk Samples

**FIGURE 2:**
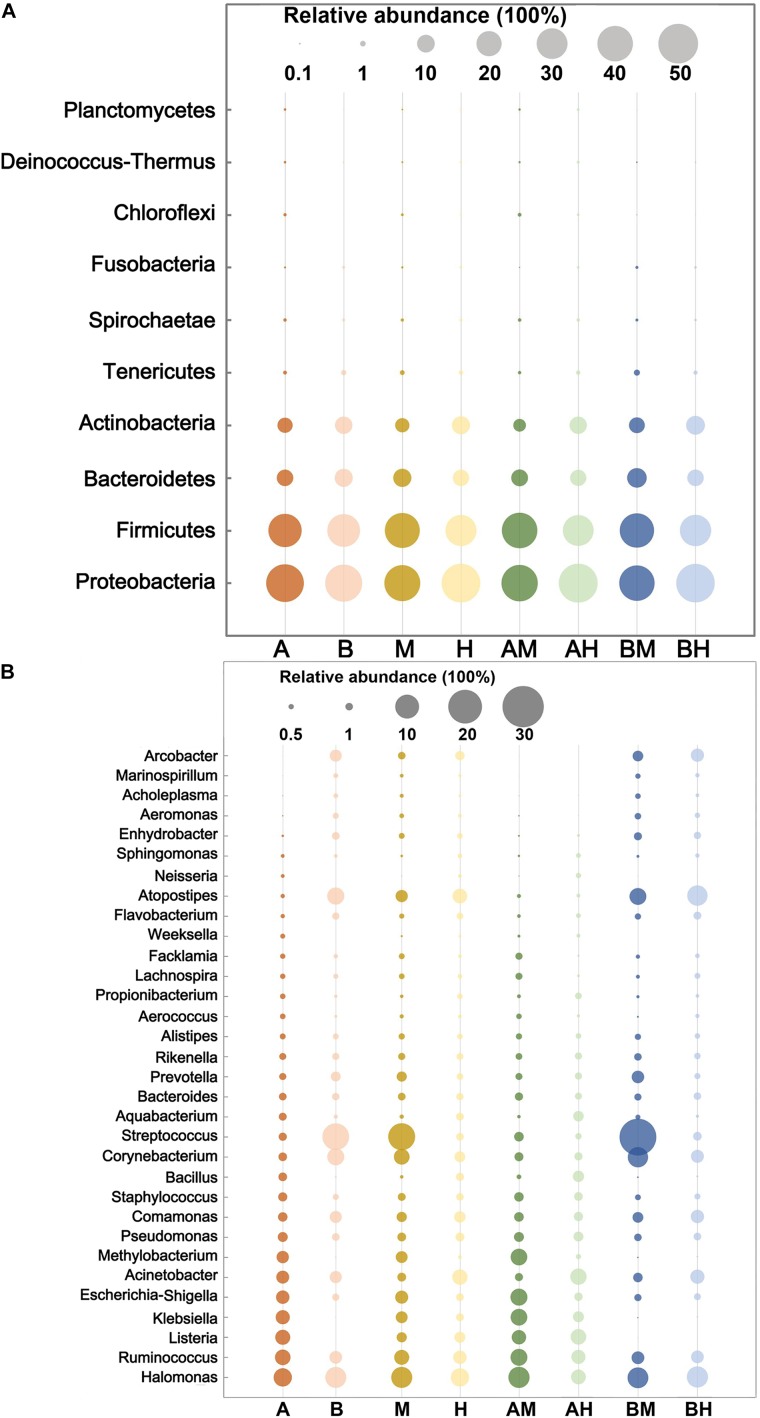
The 10 most abundant microbial phyla **(A)** and 32 genera with an abundance higher than 0.5% in at least one group **(B)**. The size of the circles indicate the relative abundances of the phylum **(A)** or genus **(B)** in each group.

The 10 most abundant microbial phyla (average abundance > 0.1%) are shown in **Figure 2A**. For each group, *Proteobacteria* was the major phylum with a prevalence that ranged from 39.96 to 48.30%, followed by *Firmicutes* (30.25–40.28%), *Bacteroidetes* (8.38–12.21%), and *Actinobacteria* (5.17–11.29%). All four phyla above were observed in all milk samples. At the genus level, a genus with a relative abundance higher than 0.5% in at least one group was defined as the predominant genus. We observed a total of 32 predominant genera as shown in **Figure 2B**. It was found that the prevalent genera were diverse in different groups. The six most prevalent microbial genera of group A were *Halomonas*, *Ruminococcus*, *Listeria*, *Klebsiella*, *Escherichia–Shigella*, and *Acinetobacter*, while the six most prevalent microbial genera of group B were *Streptococcus*, *Halomonas*, *Atopostipes*, *Corynebacterium*, *Ruminococcus*, and *Comamonas*. Some genera, such as *Halomonas* and *Ruminococcus* were prevalent in both groups A and B. When the samples were classified into groups M and H, our analysis demonstrated that *Streptococcus*, *Halomonas*, and *Corynebacterium* were the most abundant genera in group M, while *Halomonas*, *Acinetobacter*, and *Atopostipes* were the most abundant genera in group H. It was also noted that *Halomonas*, *Acinetobacter*, and *Atopostipes* were the most prevalent genera in group AM, while *Streptococcus*, *Halomonas*, and *Corynebacterium* were the most prevalent genera in group BM.

**FIGURE 3:**
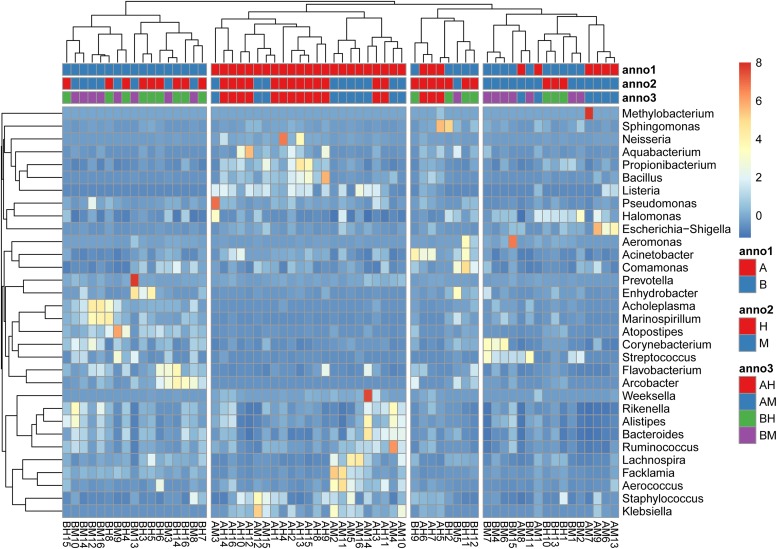
A hierarchically clustered heatmap of the microbial profiles of each milk sample. Annotations at the top of the heatmap show the clustering of milk samples. The dual hierarchical dendrogram shows the distribution of bacteria based on average linkage clustering and Euclidean distance. The color scale depicts the normalized relative abundance of each genus.

The hierarchically clustered heatmap of microbial profiles of each milk sample was generated (**Figure 3**). The prevalence of microbial genera in different samples was diverse. Some mastitic milk samples such as AM7, AM14, and BM13 were dominated by one genus, while others, especially the healthy milk samples including AH14, AH16, and BH15, showed a more balanced profile. Our results showed that 17 milk samples from group A and 23 milk samples from group B formed two distinct cluster (clades I and II), while the milk samples from groups M and H could not be completely separated. The milk samples of groups AM and AH and of groups BM and BH were also largely indistinguishable. This result suggested the microbial profiles were more similar in the milk samples collected from the same dairy farm.

### Alpha and Beta Diversities of Milk Microbiota

**FIGURE 4:**
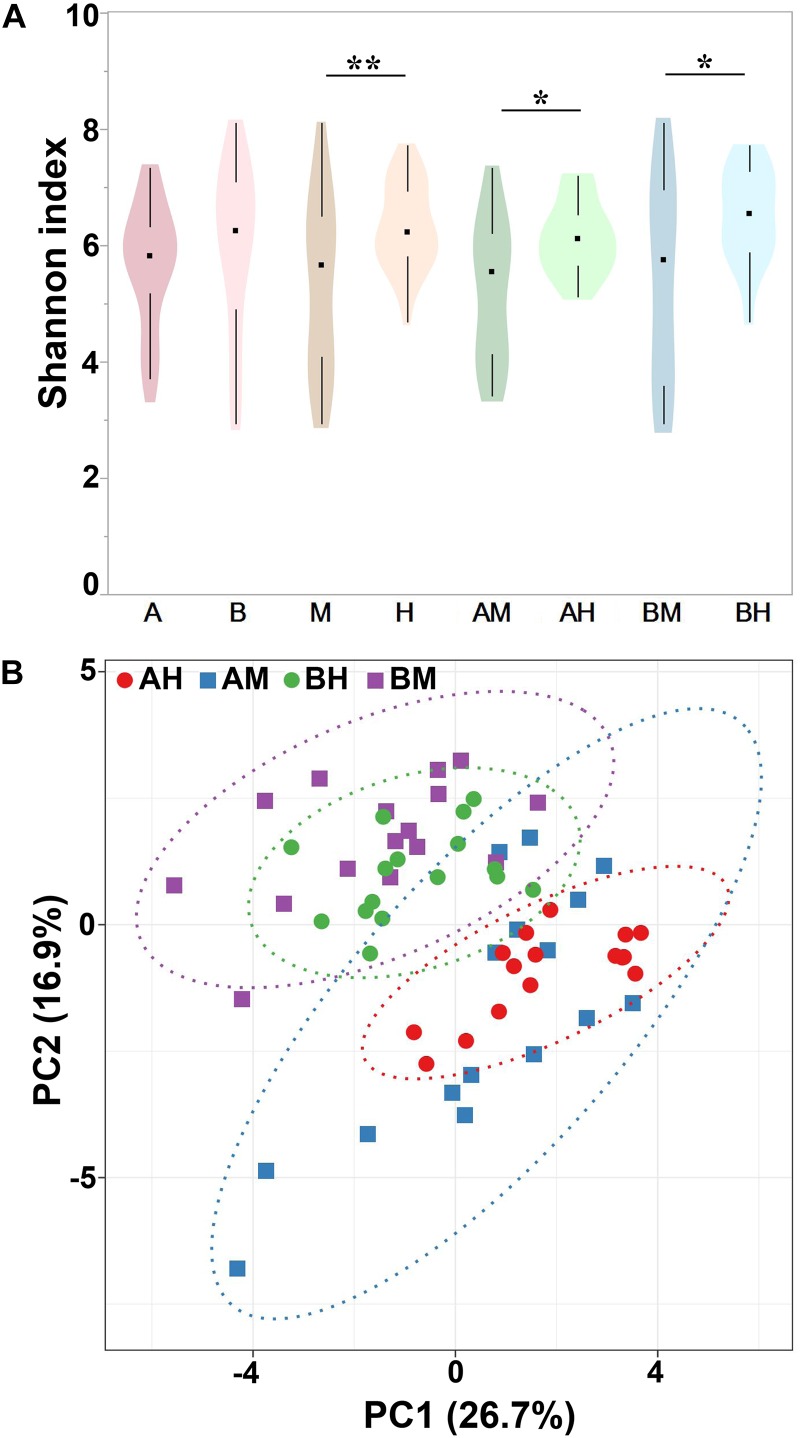
Shannon index of different groups **(A)** and PCA analysis of each milk sample **(B)**. Violin plots show the distribution of the Shannon indices of milk samples in each group, and the black points represent the medians of the Shannon indices of milk samples. ^∗^*P* < 0.05, ^∗∗^*P* < 0.01 indicates a significant difference compared between the Shannon indices of two groups.

Alpha diversity can be used to determine the microbial diversity of a given sample (Bailey et al., 2013). To analyze the microbial diversity of each milk sample, the Shannon index, which reflects the species richness and evenness, was analyzed. The violin plots showed the distribution of the Shannon indices of milk samples in each group (**Figure 4A**). We detected no significant difference between the Shannon indices of groups A and B. However, the Shannon index of group M was significantly lower than that of group H. Additionally, similar trends were found between groups AM and AH and between groups BM and BH. These results indicated that the microbial diversity of healthy groups was higher than that of the mastitis groups. Beta diversity can determine the microbial diversity between different samples (Bailey et al., 2013). To compare whole microbial composition similarities, PCA was conducted using genus-level taxonomic profiles. As shown in **Figure 4B**, the clustering of the milk samples based on their microbiota made it possible to separate samples from groups A and B. However, there was no clear separation of milk samples from groups M and H within the PCA plots. The distribution of the milk samples from group AM revealed an overlap with group AH, and a similar result was found between groups BM and BH.

### Compositional Differences Between Different Groups

**FIGURE 5:**
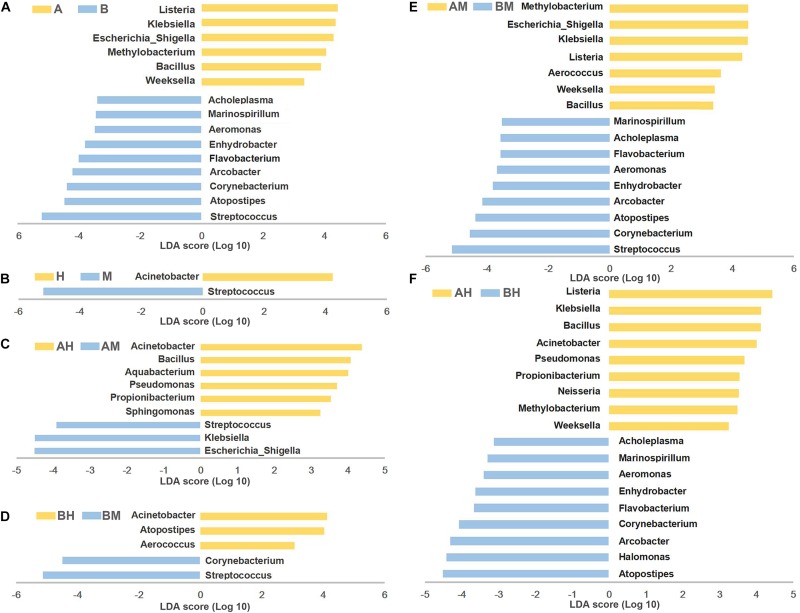
LEfSe analysis of microbiota. The microbial genera with significant differences in relative abundance compared between two groups are coded in blue and yellow as indicated in **(A–F)**. Only the genera with an LDA score > 3 (*P* < 0.05) are depicted.

To identify differences in microbial composition of milk samples between different groups, LEfSe was used to provide biomarkers at the genus-level with a linear discriminant analyses (LDA) score > 3 (*P* < 0.05). As shown in **Figure 5A**, 15 genera were statistically significantly different between group A and group B. Six genera (*Listeria*, *Klebsiella*, *Escherichia–Shigella*, *Methylobacterium*, *Bacillus*, and *Weeksella*) were significantly enriched in group A, while nine genera (*Streptococcus*, *Atopostipes*, *Corynebacterium*, *Arcobacter*, *Flavobacterium*, *Enhydrobacter*, *Aeromonas*, *Marinospirillum*, and *Acholeplasma*) were significantly enriched in group B. This result indicates that nearly half of the predominant bacterial genera were different between the two dairy farms. The comparison between groups M and H showed that *Acinetobacter* were more prevalent in group H, while *Streptococcus* were more prevalent in group M (**Figure 5B**).

To identify the biomarkers between the bacterial composition of the healthy and mastitic milk samples obtained from the same dairy farm, the comparisons were also performed between groups AM and AH (**Figure 5C**) and between groups BM and BH (**Figure 5D**). Our results showed that three genera (*Escherichia–Shigella*, *Klebsiella*, and *Streptococcus*) were identified as biomarkers in group AM, while six genera (*Acinetobacter*, *Bacillus*, *Aquabacterium*, *Pseudomonas*, *Propionibacterium*, and *Sphingomonas*) were identified as biomarkers in group AH. The comparison between groups BM and BH showed that *Acinetobacter*, *Atopostipes*, and *Aerococcus* were more enriched in group BH, while *Streptococcus* and *Corynebacterium* were more enriched in group BM. Additionally, the comparisons between groups AM and BM and between groups AH and BH were also performed. As shown in **Figures 5E,F**, the different genera identified between groups AM and BM and between groups AH and BH were similar to the different genera identified between groups A and B.

### Co-occurrence Network Analysis

**FIGURE 6:**
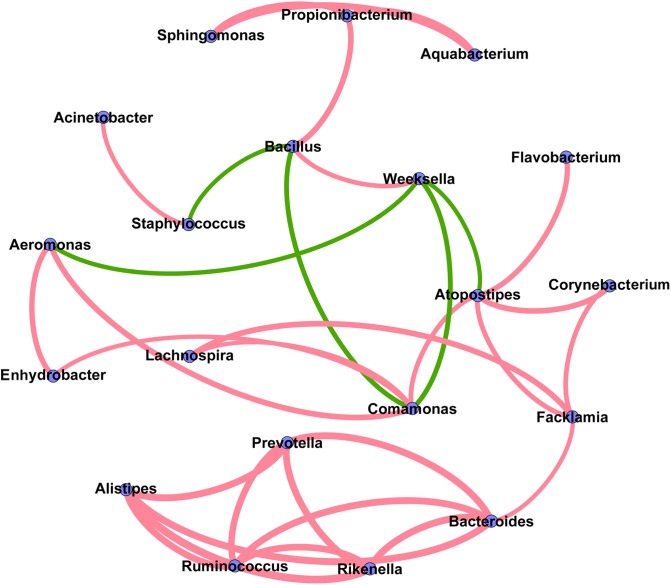
Co-occurrence network of the predominant bacterial genera in the milk samples. Each node represents one genus, and each pair of genera connected by the line has a calculated Spearman’s correlation coefficient value > 0.5 or <–0.5 (*P*-value < 0.05). Red connecting lines represent the positive significant correlations and green lines represent the negative significant correlations. The thickness of each connecting line between two nodes (edge) is proportional to the absolute value of Spearman’s correlation coefficient.

The co-occurrence network analysis was applied in order to obtain a view of the potential relationships among predominant bacterial genera in milk samples. As shown in **Figure 6**, the co-occurrence network consisted of 20 nodes and 32 edges, with 27 positive and 5 negative correlation. Interestingly, five gut-associated genera (*Prevotella*, *Ruminococcus*, *Bacteroides*, *Rikenella*, and *Alistipes*) displayed positive correlation coefficients with each other, and each individual pairing between two genera showed a strong correlation (Spearman’s correlation coefficient value > 0.8), suggesting that these genera are likely to share a symbiotic or syntrophic relationship. It was also observed that the occurrence of *Bacillus* was negatively correlated with the occurrence of *Staphylococcus* and *Comamonas*, while the presence of *Weeksella* was negatively correlated with the presence of *Aeromonas*, *Comamonas*, and *Atopostipes*. Notably, although the co-occurrence network shed light on the complex relationships of bovine milk microbiota, empirical evidences are needed to support their natural presence.

## Discussion

Bovine mastitis is a highly prevalent disease in dairy herds and it is arguably the most important disease that affects the dairy industry worldwide (Ruegg, 2017). Here, we used high-throughput 16S rRNA gene sequencing to analyze the bacterial community of milk samples collected from two dairy farms with different IRSCMs. Over the past decade, different hypervariable (V) regions of 16S rRNA including V1–V2, V1–V3, V3, V3–V4, V3–V5, V4, V4–V5, V5, V5–V6, V5–V9, V6, and V6–V8 have been sequenced to describe the microbial community (Zhang et al., 2018). For bovine milk, V1–V2 region was used frequently to describe the bacterial diversity (Oikonomou et al., 2012, 2014; Kuehn et al., 2013; Rodrigues et al., 2017). It is recognized that the selection of V regions can affect estimates on OTU richness and diversity, and the use of different V regions means that results are often not directly comparable, diminishing the value of inferences that can be drawn (Sperling et al., 2017). Therefore, to compare with the bovine milk microbiota described in previous study (Oikonomou et al., 2012, 2014; Kuehn et al., 2013; Rodrigues et al., 2017), the V1–V2 region was selected to sequence in this study. However, as has been suggested by Sperling et al. (2017), more than one marker region is needed to provide more reliable inferences in the future study.

Contrary to previous studies that only focus on the comparison between microbial composition of healthy and mastitis milk samples (Oikonomou et al., 2012, 2014; Kuehn et al., 2013), we also focused on the microbial diversity between different farms. Our study revealed strong variation between the microbiota of milk samples, with some samples clearly dominated by one genus, whereas others displayed a more balanced profile. It was also noted that the milk samples collected from two farms (A and B) could be clearly separated, while there was no clear separation of healthy and mastitis samples collected from the same farm. However, the discriminant analysis made it possible to identify the genus markers between different groups.

In this study, one of our aims was to compare the microbial community of milk samples collected from farms A and B. There were six genera more prevalent in group A and nine genera more prevalent in group B. Among the six genera more prevalent in group A, *Klebsiella* and *Escherichia–Shigella* were more enriched in group AM compared to group AH, while *Bacillus* was more enriched in group AH. The relative abundance of *Listeria*, *Methylobacterium*, and *Weeksella* showed no significant difference between groups AH and AM. Both *Klebsiella* and *Escherichia–Shigella* (particularly *Escherichia coli*), have been recognized as the pathogens that cause environmental bovine mastitis, which is caused by pathogens present in the digestive tract of cows or their surroundings (Schukken et al., 2012). Contrarily, *Bacillus* was found to be more prevalent in group AH than group AM. In addition, our co-occurrence analysis showed there was a negative correlation between *Bacillus* and *Staphylococcus*, although this correlation had a weak coefficient value (0.51). These results suggest that *Bacillus* may contribute to the overall health of cows, however, which *Bacillus* species would be beneficial and its role warrants further investigation.

Although the 16S rRNA sequencing in our study was not insufficient for accurate taxonomic assignment at the species level, the percentage of sequences assigned to the level of species could range from 31.6 to 36.8% in different group. Our result showed that the sequences, which were assigned to *Listeria* at the genus level, were all assigned to *Listeria monocytogenes* at the species level. To determine which kind of *Listeria* species were present in farm A, we tried to isolate *Listeria* from 156 milk samples newly collected from farm A according to the previous study (Osman et al., 2016). Finally, 16 *L. monocytogenes* strains (positive rate 10.26%), but no *L. innocua* and *L. ivanovii*, were isolated from farm A. Thus, the *Listeria* sequences obtained from farm A in this study should be classified as *L. monocytogenes* at the species level. Bovine mastitis caused by *L. monocytogenes* is rare (Osman et al., 2016), in addition, the cases of short-live excretion of *L. monocytogenes* bacteria in milk samples do not show any symptoms, and cases of prolonged mastitis due to *Listeria* are not reported (Winter et al., 2004). However, *L. monocytogenes* has been involved in several outbreaks of listeriosis, occurring after consumption of contaminated milk and milk products worldwide (El Marnissi et al., 2016). Our study indicated that cows can be healthy reservoirs of *L. monocytogenes*, thus, there is a need for continued surveillance for the presence of *Listeria* in bovine milk. The *Methylobacterium* genus includes a group of strictly aerobic, Gram-negative bacteria which are ubiquitous and detected in soil, freshwater, and lake sediments (Bracke et al., 2014). These bacteria have been reported to cause opportunistic infections in immunocompromised hosts (Sanders et al., 2000). Recently, *Methylobacterium* was also observed as a contaminant in milk samples (Bracke et al., 2014), but no reports have shown a link to bovine mastitis. The genus *Weeksella* includes only one species, *Weeksella virosa*, that has been isolated from human clinical specimens (Sankar et al., 2015). Slenker et al. (2012) also described a fatal case of *W. virosa* sepsis in a young female with end-stage renal disease. However, similar to *Methylobacterium*, no research studies have reported a link between *Weeksella* and bovine mastitis.

Among the nine predominant genera more prevalent in group B, the relative abundances of *Streptococcus* and *Corynebacterium* were significantly higher in group BM, while *Atopostipes* was significantly higher in group BH. The relative abundances of six other genera (*Arcobacter*, *Flavobacterium*, *Enhydrobacter*, *Aeromonas*, *Marinospirillum*, and *Acholeplasma*) were similar between groups BH and BM. The *Streptococcus* genera, including *Streptococcus agalactiae*, *Streptococcus* *uberis*, and *Streptococcus dysgalactiae*, are well-known mastitis-causing pathogens (Klaas and Zadoks, 2017; Pang et al., 2017). *Corynebacterium* has also been identified as the pathogen associated with mastitis in dairy cows, often being described as contagious (Oultram et al., 2017). Previous studies reported that *Corynebacterium* could be detected in bulk tank milk samples collected from 894 China dairy herds at a frequency of 17.0% (Bi et al., 2016) and from 1242 dairy cows in Brazil at a frequency of 22.9% (Goncalves et al., 2016). In addition, in the microbiome of bulk tank milk, *Streptococcus* and *Corynebacterium* were encountered in significantly higher relative abundances in the HSCC (high somatic cell count) group when compared with the LSCC (low somatic cell count) (Rodrigues et al., 2017). Thus, we speculated that *Streptococcus* and *Corynebacterium* may have been responsible for the higher IRSCM in farm B.

In previous studies, *Atopostipes* isolated from pig manure was proposed as a new genus in 2004 (Samanta et al., 2015) and has recently been identified as a pig-specific fecal indicator (Jeong et al., 2011). *Atopostipes* is also found in the microbial community of the outer udder skin of calves (Yeoman et al., 2018). In addition, *Atopostipes* was exclusively present in feces and intestinal tissue of EAE (experimental autoimmune encephalomyelitis)-resistant rats (Stanisavljevic et al., 2016). However, *Atopostipes* has never been linked to bovine health or the safety of milk. During recent years, *Arcobacter* has emerged as an important foodborne and waterborne zoonotic pathogen worldwide and has been classified as a serious hazard to human health (Ramees et al., 2017). The presence of *Arcobacter* has been observed in cow milk (Yesilmen et al., 2014), cow feces (Van Driessche et al., 2005), and in milk filters in a water buffalo dairy farm in Italy (Serraino et al., 2013). In addition, *Arcobacter* has been isolated from a dairy herd that underwent an outbreak of mastitis (Logan et al., 1982). However, although *Arcobacter* has been associated with reproduction disorders and mastitis in livestock, it has also been isolated from healthy animals frequently (De Smet et al., 2011). Similarly, our results showed that *Arcobacter* was prevalent in farm B, but there was no significant difference between groups BM and BH.

Except for the genera discussed above, our study found no significant difference in the prevalence of 17 predominant genera between the two farms. Interestingly, five predominant genera (*Prevotella*, *Ruminococcus*, *Bacteroides*, *Rikenella*, and *Alistipes*), which were prevalent in all milk samples, were all typically gut-associated genera (Huws et al., 2011). Previous studies have also shown that *Prevotella* (Oikonomou et al., 2012, 2014), *Ruminococcus* (Oikonomou et al., 2012, 2014; Kable et al., 2016; Oultram et al., 2017), *Bacteroides* (Oikonomou et al., 2012, 2014; Oultram et al., 2017), and *Alistipes* (Gschwendtner et al., 2016) could be detected in cow milk. In addition, our study showed these five genera had strong positive correlation coefficients with each other. These findings suggest that gut bacteria can be one of the predominant microbial contamination in milk. The gut bacteria in milk can be contaminated from the herd feces (Doyle et al., 2017b). Alternatively, as described by several authors (Addis et al., 2016), the gut bacteria would also reach the mammary gland through an endogenous entero-mammary pathway. However, these gut-associated genera may not contribute to the appearance of bovine mastitis since there were no significant differences between their relative abundances in each group. *Halomonas* was identified as the most prevalent genus in both farms A and B. *Halomonas*, as a negative bacterium, has been detected in Danish raw milk cheeses (Masoud et al., 2011) and is found to be the most frequent OTU in short-ripened cheeses (Schornsteiner et al., 2014). As speculated by Ishikawa et al., *Halomonas* in dairy products originated in marine environments and was introduced via the sea salt added to cheese surfaces during washing and dry salting (Ishikawa et al., 2007). However, our study indicated that *Halomonas* is a frequent contaminant of milk microbiota, thus, the *Halomonas* in dairy products may come directly from raw milk.

In this study, we also focused on the bacterial genera which were more prevalent in group M and group H to identify the mastitis-associated pathogens and the potential beneficial bacteria present in both dairy farms. *Streptococcus* was identified as the core mastitis-associated pathogen in both farms A and B, while *Acinetobacter* was more prevalent in group H. In the assessment of the human milk microbial community, healthy controls possessed relatively more *Acinetobacter* in comparison to the mastitic group (Patel et al., 2017). It was also reported that *Acinetobacter* is a member of the core milk microbiota (Kable et al., 2016) and is frequently detected in raw milk (Quigley et al., 2013). Although the role of *Acinetobacter* in milk is still unknown, it is well documented that *Acinetobacter* is frequently associated with antibiotic resistance and human clinical infections (Dadar et al., 2012). Recently, it has been shown that *Acinetobacter* strains isolated from raw milk also exhibited antibiotic resistance (Gurung et al., 2013). Thus, although *Acinetobacter* may be important for the healthy status of cows, it is needed to examine the antibiotic resistance and genetic characteristics of *Acinetobacter* strains in milk samples since they could be a public health concern.

Additionally, the microbial diversity was also compared between groups AH and AM, and between groups BH and BM. Our study indicated that *Klebsiella*, *Escherichia–Shigella*, and *Streptococcus* were the pathogens that caused mastitis in farm A, while *Streptococcus* and *Corynebacterium* were the mastitis-causing pathogens in farm B. These genera are all well-recognized mastitis pathogens. It was also observed that *Propionibacterium* was identified as a biomarker in group AH. Previous studies have shown that *Propionibacterium* was present in all milk samples obtained from healthy quarters of bovine (Oikonomou et al., 2014), and the abundance of *Propionibacterium* is negatively correlated with the total bacterial count in cow milk (Li et al., 2018). In addition, *Propionibacterium* was only identified in the teats of non-infected quarters of dairy cows by denaturing gradient gel electrophoresis (DGGE) (Braem et al., 2012). These studies indicate that *Propionibacterium* may be potential beneficial bacteria for cows and warrant further investigation into its role in milk.

In our study, it became apparent that the 16S rRNA gene sequencing approach can be very helpful for elucidating the microbiota of milk. In conclusion, our study found that the relative abundances of the pathogens *Streptococcus* and *Corynebacterium* in farm B were 15.74 and 6.38%, respectively, while the relative abundances of pathogens *Klebsiella*, *Escherichia–Shigella*, and *Streptococcus* in farm A were 4.41, 3.95, and 1.46%, respectively. The relative abundance of all pathogens in farm B (22.12%) was higher than that of all pathogens in farm A (9.82%). Of note, although *Streptococcus* was present in both farms A and B, the relative abundance of *Streptococcus* in farm B (15.74%) was significantly higher than that in farm A (1.46%). In contrast, the genera *Bacillus* were more enriched in both groups A and AH. These results may helpful for explaining the lower IRSCMs in farm A compared to farm B. Additionally, the genera including *Halomonas* and the gut-associated genera *Prevotella*, *Ruminococcus*, *Bacteroides*, *Rikenella*, and *Alistipes* were identified as the commensal bacteria prevalent in all milk samples. Moreover, we also found that *L. monocytogenes* was enriched in group A, suggesting there is a need for surveillance of the milk microbiota.

Admittedly, while 16S rRNA gene sequencing is a powerful approach to describe bacterial community and leads to the discovery of many unexpected evolutionary lineages, certain limitations do have to be considered (Salter et al., 2014; Oultram et al., 2017). These limitations, including choices relating to sample collection, sample storage and preservation, DNA extraction method, contaminating microbial, amplifying primers, sequencing technology, read length and depth and bioinformatics analysis techniques, can affect the OTU richness and diversity (Salter et al., 2014). However, to overcome these limitations, many efforts have been made to evaluate the influence of sampling technique (Metzger et al., 2018), sample storage condition (Doyle et al., 2017a) and DNA extraction methods (Lima et al., 2018), design new amplifying primers (Bahram et al., 2018), remove bacterial DNA contamination (Karstens et al., 2018), develop sequencing technology (Tedersoo et al., 2018) and bioinformatic analysis techniques (Gasc and Peyret, 2018). Recently, the full-length 16S rRNA sequencing technology (Pootakham et al., 2017) and method (Fuks et al., 2018) have been demonstrated to be useful for the accurate classification of the bacterial community composition at the species level. As 16S rRNA sequencing becomes cheaper and faster toperform, and with new technology developing, it is likely to become a cost-effective and more reliable approach to determine microbial community.

Overall, our study revealed complex diversity between the microbial communities of dairy farms with different IRSCMs. This suggests that varying IRSCMs may not only be determined by the heterogeneity and prevalence of mastitis-causing pathogens but also related to the potential beneficial bacteria. Further studies could therefore aim to sequence more samples collected from more dairy farms with different IRSCMs to determine the relationship between milk microbiota and IRSCMs. Here, we encourage that the milk microbiota be examined for bovine mammary gland health management, which can provide complementary information such as the raw milk microbial ecology, and the detection of fastidious bacteria and polymicrobial infections. The metataxonomic approach, which could be combined with the bacterial culture or targeted specific real-time PCR, would not only be helpful for establishment of a targeted mastitis control system but also for the control of the safety and quality of dairy products. This would not only be helpful for establishment of a targeted mastitis control system but also for the control of the safety and quality of dairy products.

## Ethics Statement

The sampling method was approved by the Animal Care and Ethics Committee of Jiangsu Academy of Agricultural Sciences (SYXK2015-0020).

## Author Contributions

MP and RaW designed the experiments. MP, HB, LS, TH, HaZ, YZ, HuZ, LZ, and RuW performed the experiments. MP, XX, LS, and TH analyzed the data. MP, XX, and RaW drafted the manuscript. KX and RaW coordination of research. All authors read and approved the final manuscript.

## Conflict of Interest Statement

The authors declare that the research was conducted in the absence of any commercial or financial relationships that could be construed as a potential conflict of interest.
